# Octreotide plus IBI-318 plus anlotinib in the treatment of multiple neuroendocrine metastases of unknown primary lesions: a case report

**DOI:** 10.3389/fonc.2024.1390299

**Published:** 2024-12-11

**Authors:** Haoyue Qin, Huan Yan, Xing Zhang, Zhe Huang, Yangqian Chen, Yuda Zhang, Siqi Xiang, Yongchang Zhang, Nong Yang, Liang Zeng

**Affiliations:** ^1^ Graduate Collaborative Training Base of Hunan Cancer Hospital, Hengyang Medical School, University of South China, Hengyang, China; ^2^ Department of Medical Oncology, Lung Cancer and Gastrointestinal Unit, Hunan Cancer Hospital/The Affiliated Cancer Hospital of Xiangya School of Medicine, Central South University, Changsha, China; ^3^ The Cancer Center, The Second People's Hospital of Hunan Province/The School of Clinical Medicine, Hunan University of Chinese Medicine, Changsha, China

**Keywords:** octreotide, IBI-318, neuroendocrine tumors, metastases, anlotinib

## Abstract

**Background:**

The second-line treatment of neuroendocrine tumors (NETs) of unknown primary origin remains uncertain. This report presented a patient who received octreotide plus IBI-318 plus anlotinib as a second-line treatment for multiple metastatic NETs of unknown primary lesions after the failure of octreotide plus everolimus.

**Case presentation:**

A 32-year-old male patient presented with elevated CEA (197.83 ng/ml) without specific symptoms. A contrast-enhanced computed tomography (CT) scan showed multiple metastatic lymph nodes and multiple low-density nodules in the liver of undetermined nature. A right supraclavicular lymph node biopsy indicated NET, but the primary tumor origin remained unknown. PD-L1 expression was negative in tumor tissue according to immunohistochemistry. Immunofluorescence indicated the CD4^+^ T cells, CD8^+^ T cells, and Treg cells were gathered around blood vessels, with only a few infiltrating lymphocytes in the tumor tissue. Treatment with octreotide (30 mg/28 d) plus everolimus (5 mg qd) led to disease progression after three cycles. Treatment was changed to octreotide (30 mg/28 d) plus IBI318 (400 mg/28 d) plus anlotinib (10 mg/1-14 d/q3w), leading to partial remission, which was sustained up to the last follow-up (June 20, 2023), with a PFS of 11 months. The patient experienced no treatment-related adverse reactions.

**Conclusions:**

Octreotide plus IBI318 plus anlotinib achieved benefits in a patient with advanced NETs of unknown primary lesions after first-line treatment failure, even though with low PD-L1 expression. This case suggests that combining SSAs, TKIs and PD-1/PD-L1 inhibitors could be an alternative second-line treatment for patients with advanced, well-differentiated NETs.

## 1 Introduction

Neuroendocrine tumors (NETs) are rare, slow-growing neoplasms of neuroendocrine origin (1–3). The disease is often asymptomatic and detected incidentally, but it may present with symptoms that are related to excessive hormone release (1, 2). NETs meet the criteria for an orphan disease in the United States of America (USA), and their estimated incidence is 6.98 per 100,000 person-years (1, 4). Common types of neuroendocrine tumors include medullary thyroid cancer, gastrointestinal neuroendocrine tumors, etc., but the primary site is unknown in some cases (1–3). The median overall survival (OS) of patients with NETs is 9.3 years, but the actual individual survival fluctuates significantly due to stage, grade, age at diagnosis, primary site and time period of diagnosis (4). Still, the 5-year survival rates of patients with metastatic NETs are 1.7%-10.7%, depending upon the type of NET (5).

Patients with advanced NETs of unknown primary origin are treated with systemic therapies, including somatostatin analogs (SSAs), mTOR inhibitors, multi-kinase inhibitors, etc. (3, 6). Long-acting octreotide and lanreotide hydrogels, as common SSAs can exert antitumor effects by binding to the somatostatin receptor (SSTR), and SSAs are recommended as first-line treatment for advanced, SSTR-positive, slow-growing gastroenteropancreatic NETs (GEP-NETs) with a Ki-67 index of ≤10% and for NETs of unknown primary lesions (7, 8). Everolimus, an orally administered mTOR inhibitor, has exhibited a capacity to postpone tumor advancement in patients with gastroenteropancreatic, pulmonary NETs and NETs of unknown primary origin (9, 10). In the phase III SANET-p and SANET-ep trials, the new tyrosine kinase inhibitor (TKI) surufatinib prolonged the progression-free survival (PFS) of pancreatic and extrapancreatic NETs (11, 12). Thus, it is recommended for the treatment of pancreatic and extrapancreatic NETs.

Immune checkpoint inhibitors (ICIs) represent a significant shift in the cancer treatment paradigm, and they can be combined with anti-angiogenesis targeted drugs (13, 14). A previous meta-analysis showed that ICIs as monotherapy had low objective response rates (ORRs), and combination therapy might improve the ORRs in neuroendocrine neoplasms (15). Various ICIs and antiangiogenic agents are available, and the optimal combination regimen remains to be explored for neuroendocrine neoplasms.

Anlotinib is a novel oral antiangiogenic multi-target TKI that targets VEGFR-1-3, FGFR1-4, PDGFR-α/β, c-Kit, and Ret, inhibiting angiogenesis and tumor growth (16). Anlotinib can improve the PFS in patients with medullary thyroid cancer (17). IBI-318 is an anti-PD-1/PD-L1 bispecific antibody and a phase I trial suggested its efficacy and safety in patients with solid tumors (18). There are two reports of anlotinib combined with ICIs for esophageal NETs (19, 20), but the efficacy of the combination treatment in patients with unknown primary lesions is unclear.

We reported a patient who received octreotide plus IBI-318 plus anlotinib as a second-line treatment for multiple metastatic NETs of unknown primary lesions after the failure of octreotide plus everolimus.

## 2 Case report

**Figure 1:**
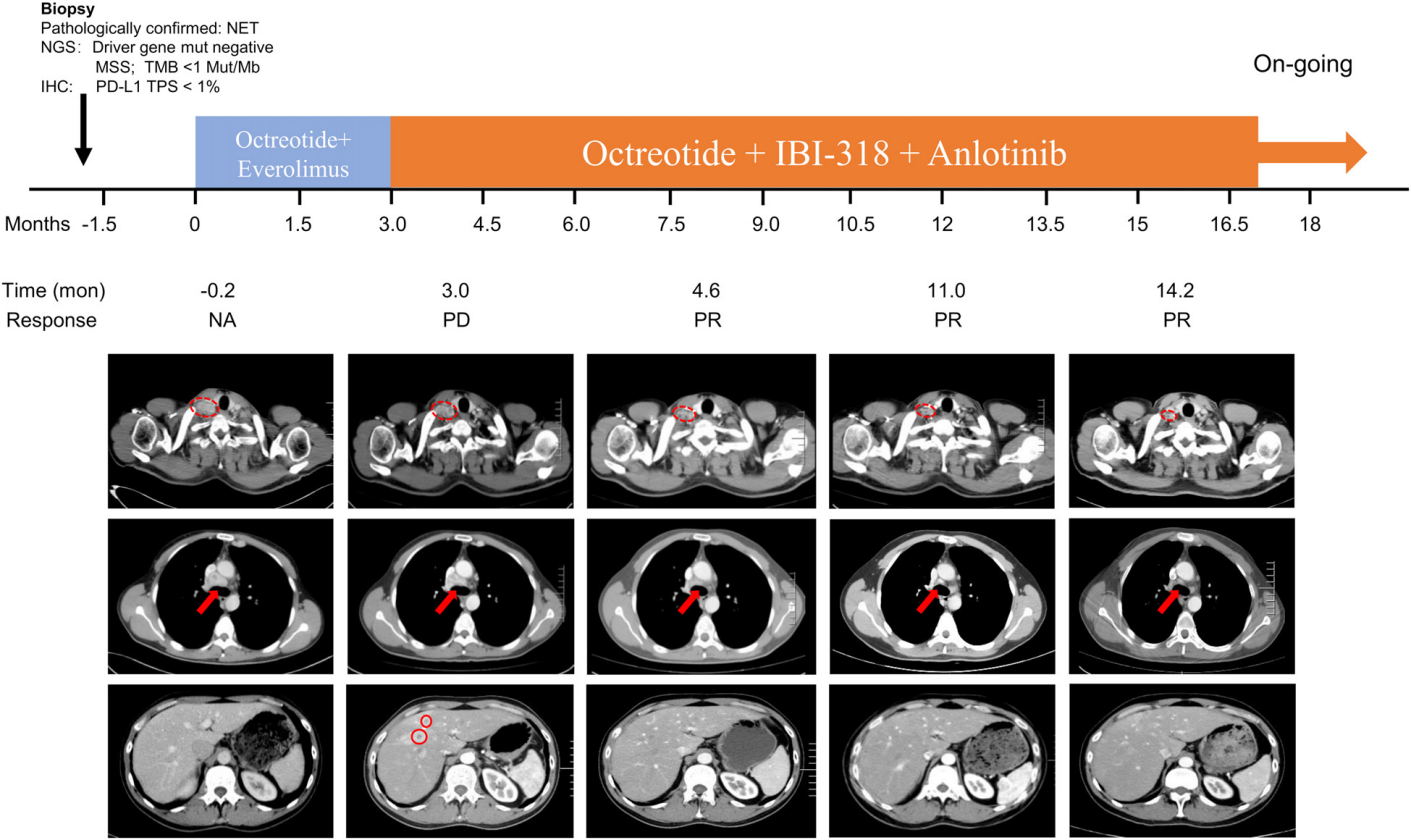
Timeline of the medication and computed tomography (CT) results before and during treatment. A pre-treatment enhanced CT scan was performed on April 11, 2022, showing multiple swollen lymph nodes in the right lower neck, bilateral supraclavicular areas, and left internal mammary area and mediastinum, and multiple low-density nodules in the liver. The first enhanced CT after starting treatment was on July 12, 2022, and showed that the multiple swollen lymph nodes in the right lower neck, bilateral supraclavicular areas, left internal mammary area, and mediastinum were smaller than before treatment, and multiple new ring-enhanced nodes appeared in the liver. The second enhanced CT scan after starting treatment was on August 30, 2022, and showed that the enlarged lymph nodes and liver nodules were smaller. The third enhanced CT scan after starting treatment was performed on March 15, 2023, and showed that the lymph nodes and liver lesions were stable. The fourth enhanced CT scan on June 20, 2023, showed that the lymph nodes were slightly smaller, while the liver lesions were roughly stable.

**Figure 2:**
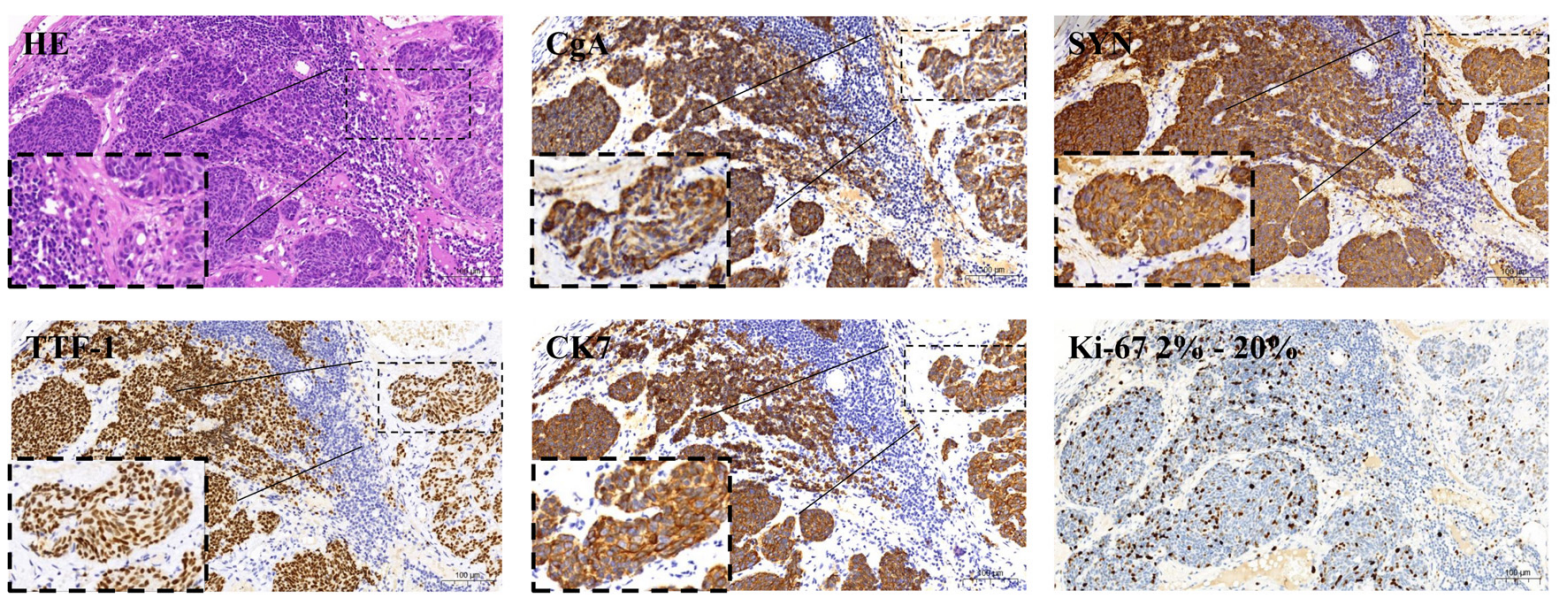
Tumor immunohistochemistry results before treatment: positive expression of chromogranin A (CgA), positive expression of synaptophysin (Syn), positive expression of cytokeratin 7 (CK7), positive expression of thyroid transcription factor 1 (TTF-1), and a Ki-67 proliferation index about 15%, directed at the individual tumor samples examined.

**Figure 3:**
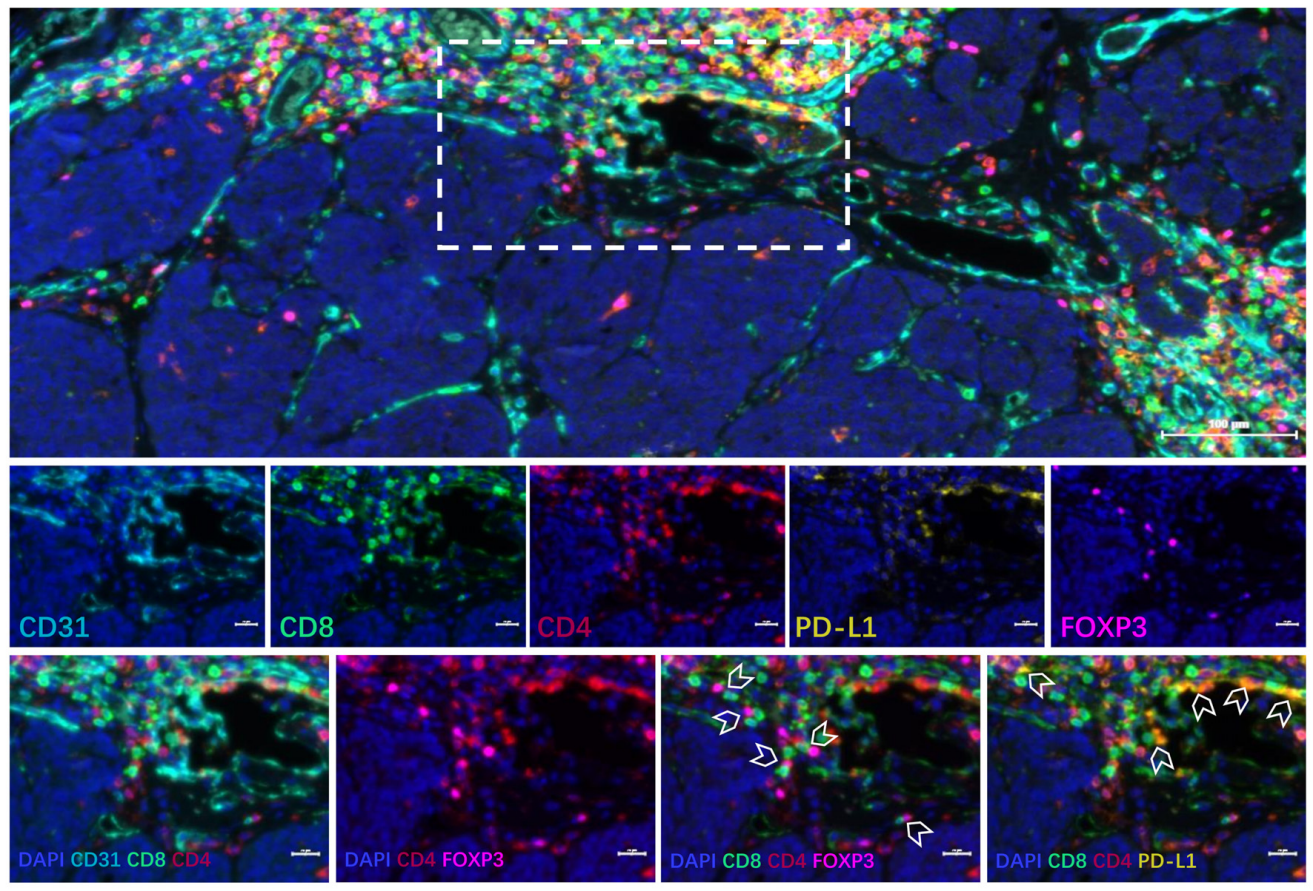
Immunofluorescence staining results of tumor tissue before treatment. DAPI, CD31, and CD8/CD4 suggested that T cells accumulated around blood vessels, and there were few infiltrating lymphocytes in the tumor parenchyma area. DAPI, CD8, and forkhead box P3 (FOXP3) suggested that Treg cells accumulated around blood vessels. DAPI, CD8, CD4, and FOXP3 suggested that Tregs were mixed with CD8 cells. DAPI, CD8, CD4, and programmed cell death ligand 1 (PD-L1) suggested that some CD4 and CD8 cells expressed PD-L1.

The patient was a 32-year-old male. A physical examination on January 14, 2022, revealed elevated CEA (197.83 ng/ml) without specific symptoms. The patient had a 5-year history of smoking and no history of alcohol consumption. In addition, he denied any family history of malignant tumors. The physical examination findings of the patient showed multiple enlarged lymph nodes in the neck with tenderness and no hepatosplenomegaly. A contrast-enhanced computed tomography (CT) scan of the chest, pelvis, and abdomen (April 11, 2022) showed 1) multiple enlarged lymph nodes in the right lower neck, bilateral supraclavicular area, left internal mammary area, and mediastinum, 2) multiple low-density nodules in the liver of undetermined nature, and 3) no abnormalities in bilateral lungs (**Figure 1**, **Table 1**). The ^18^F-FDG positron emission tomography (PET)/CT conducted on April 12, 2022, showed that the bilateral neck and supraclavicular areas, mediastinum (1R, 2R, 3A, and 4R groups), and internal mammary lymph nodes were enlarged and had a slightly increased FDG metabolism, suggesting lymph node metastases. The ^68^Ga-PET/CT conducted on April 13, 2022, showed multiple lymph nodes in the bilateral neck, bilateral supraclavicular area, anterior mediastinum (2R and 4R groups), and left internal mammary area, with increased expression of somatostatin receptor (SSTR2/5). A right supraclavicular lymph node biopsy was performed on February 16, 2022, which showed that the pathological type was metastatic neuroendocrine tumor, G2. The immunohistochemistry results included CK+, TTF-1+, NapsinA-, Syn+, CgA+, CK7+, P63-, P40-, ALK-, SSTR2(focus+), SSTR5(+++), Ki67 of 15% (**Figure 2**), and PD-L1 TPS <1%. The patient was diagnosed with multiple metastatic non-functioning NET (G2) of unknown primary origin, stage IV. Next-generation sequencing (NGS) of tumor tissue demonstrated negative mutation in driver genes, microsatellite stable (MSS), and tumor mutation burden (TMB) <1 mut/Mb. Immunofluorescence showed that lymphocytes were gathered around blood vessels, few lymphocytes infiltrated the tumor parenchyma area, and Treg cells gathered around blood vessels. PD-L1 protein expression was low (**Figure 3**).

**Table 1:** The timeline of treatments and responses.

Time point	Measures
February 2022	Diagnosed with multiple metastatic non-functioning neuroendocrine tumor (G2) of unknown primary origin, stage IV.
April 14, 2022	Started 3 cycles of long-acting octreotide microspheres (30 mg/28 d) plus everolimus (5 mg/qd).
July 12, 2022	Disease progression observed.
July 15, 2022	Treatment changed to long-acting octreotide microspheres (30 mg/28 d) plus IBI318 (400 mg/28 d) plus anlotinib (10 mg/1-14 d/q3w).
August 30, 2022	Partial disease remission noted.
March 16, 2023	Continued partial disease remission.
June 20, 2023	Continued partial disease remission.

Treatment with long-acting octreotide microspheres (30 mg/28 d) plus everolimus (5 mg/qd) was started on April 14, 2022. In June 2022, the patient felt pain in the mediastinal area of the chest and internal mammary lymph node areas. An evaluation was conducted after three cycles, and an enhanced CT conducted on July 12, 2022, showed that 1) the lymph nodes in the right lower neck, bilateral supraclavicular area, left internal mammary area, and mediastinum were reduced compared with baseline, 2) multiple new ring-shaped enhancing nodules were observed in the liver, suggesting metastases, and 3) no abnormalities in bilateral lungs. Hence, disease progression (PD) was revealed (**Figure 1**, **Table 1**).

For G2 NETs with an unknown primary origin, after first-line treatment with long-acting octreotide or everolimus, there is currently no standard second-line treatment model, and exploration is still ongoing. Anti-angiogenic TKIs (such as surufatinib) have achieved encouraging therapeutic effects in the treatment of NETs (11, 12), and the synergistic enhancement of anti-angiogenic drugs combined with ICIs has been verified in a large number of basic and clinical studies (19, 21). Therefore, on July 15, 2022, treatment was changed to long-acting octreotide microspheres (30 mg/28 days) plus IBI-318 (400 mg/28 days) plus anlotinib (10 mg/day, 1-14 days/cycle). IBI-318 was provided for compassionate use. The treatment regimen of octreotide plus IBI-318 plus anlotinib was not part of a clinical trial but was administered based on clinical judgment and the availability of these drugs. The patient highly recognized the concept of synergistic enhancement of anti-tumor activity with anti-angiogenic drugs plus ICIs and was willing to try this kind of regimen. CT was performed regularly during treatment. On August 30, 2022, CT showed that the lymph nodes in the right lower neck, bilateral supraclavicular area, left internal mammary area, and mediastinum and the liver lesions were smaller, indicating partial disease remission (**Figure 1**, **Table 1**). The following CT indicated a relatively stable disease. After changing treatment, the patient’s CEA level decreased to 60.56 ng/ml and the pain was also relieved. The patient was followed up until June 20, 2023, and was still in partial remission (**Figure 1**, **Table 1**), with a PFS of 11 months. As of June 20, 2023, the patient experienced no treatment-related adverse reactions.

## 3 Discussion

The case reported here was a 32-year-old male with multiple metastases from a non-functioning NET (G2) of unknown primary origin, stage IV. Disease progression occurred after three cycles of treatment with octreotide plus everolimus. The treatment regimen was changed to octreotide plus IBI318 plus anlotinib, achieving a partial remission sustained until the last follow-up 11 months after starting the second-line treatment.

In the case reported here, the SSTR2/5 expression was increased, and no driver genes mutation were detected. According to the NCCN guidelines (version 1.2022) (22), SSAs (when the NETs are SSTR-positive), everolimus, peptide receptor radionuclide therapy (PRRT), capecitabine with temozolomide, and cisplatin with etoposide can all be used as first-line treatment regimens for G1 or G2 NETs with unknown primary lesions. The phase III RADIANT-4 trial showed that patients with NETs of unknown primary lesions treated with everolimus had a median PFS of 13.6 (95% confidence interval (CI): 9.2-17.3) months, compared with 5.4 (95%CI: 3.6-9.3) months for the placebo (hazard ratio (HR)=0.56, 95%CI: 0.37-0.84), indicating that everolimus can be used for the treatment of NETs with unknown primary lesions (23). Therefore, the patient reported here was first treated with octreotide plus everolimus, but disease progression was observed after three treatment cycles. Octreotide has a high affinity for SSTR2 and SSTR5 (24); currently, SSAs such as octreotide and lanreotide have shown to be beneficial for the symptomatic and biochemical improvement of patients with NETs and exhibit a good safety profile (25). A systematic review including 17 studies indicated that high doses of long-acting octreotide (≥30 mg/month) are commonly used in the treatment of NETs (26). Although the use of SSAs typically requires the tumor to be SSTR-positive, SSTR positivity does not predict treatment response (27). Additionally, SSAs appear to be more effective in NETs with Ki-67 ≤10%, especially ≤5% (27). Considering the Ki-67 expression in this case is 15%, it seems understandable that the treatment was ineffective in this patient.

Following first-line treatment failure, the NCCN guidelines (version 1.2022) recommended alternative first-line drugs. In other words, the second-line treatment of NETs of unknown primary origin remains uncertain, primarily because there are limited evidences from clinical trials on second-line treatment for NETs of unknown primary lesions. In clinical practice, for patients with NET G1/G2 and Ki-67 < 10% whose tumors are observed to progress rapidly while on SSA treatment, there is a tendency to choose peptide receptor radionuclide therapy (PRRT) as a second-line treatment (28). However, due to the radioactivity involved, PRRT has accessibility issues and is not available in every hospital. In addition, PRRT is also more expensive, with poor cost-effectiveness. Besides, in this case, the patient’s Ki-67 is 15%, therefore, targeted therapy is preferred as the first choice for second-line treatment (29). For patients with well-differentiated pancreatic NETs who have failed first-line treatment, the type of second-line treatment is significantly associated with patient treatment outcomes. The median PFS for patients receiving PRRT, targeted therapy, high-dose SSA, or switching to another SSA, and chemotherapy as second-line treatment were 26 months, 16 months, 10 months, and 7.7 months, respectively (30). A retrospective cohort study analyzed the PFS and OS of patients with advanced well-differentiated enteropancreatic G1-G3 who failed SSA treatment and received PRRT compared to those who received targeted therapy or chemotherapy. It suggested that the median PFS of patients receiving PRRT as second-line treatment was 2.2 years, which was significantly higher than that of patients receiving targeted therapy or chemotherapy as second-line treatment (0.6 years), with no significant difference in OS between the two groups (31). However, the aforementioned studies were all conducted on pancreatic-origin NETs, and the prognosis of these patients is generally better than that of patients with NETs of unknown origin (4). Therefore, it is difficult to compare the treatment outcomes of this patient with the results of the aforementioned studies.

PD-1/PD-L1 inhibitors have been approved for the treatment of various cancers (13, 14, 32), but the evidence for the use of ICIs in NETs remains thin. Ozdirk et al. (33) reported eight patients with NETs in whom pembrolizumab, avelumab, or nivolumab plus ipilimumab was used as salvage treatment, leading to partial remission in three patients and stable disease in one. In 2022, Gubbi et al. (34) published a review suggesting that ICIs can be an option for NETs but that there was no clear efficacy or safety benefit compared with other systemic therapies. On the other hand, Weber & Fottner (35) found that ICIs could be particularly useful in NETs with high tumor burden, microsatellite instability, and/or high mutational load. The patient reported here had a high tumor load but displayed microsatellite stability and a low tumor mutation burden. In addition, PD-L1 expression was negative in tumor tissue. The CD4+ T cells, CD8+ T cells, and Treg cells were gathered around blood vessels, with only a few infiltrating lymphocytes in the tumor tissue. Therefore, the patient had a “cold tumor,” which is typically not responsive to treatments with ICIs (36). However, combination therapy may improve tumor sensitivity to ICIs.

Currently, anti-angiogenic drugs, including antibodies targeting the VEGF-VEGFR pathway and TKIs, are one of the few combination therapies proven to enhance the efficacy of ICIs (37). VEGF can induce local immunosuppression in tumors through various mechanisms (38–40). Preclinical studies have shown that anti-angiogenic drugs can promote vessel normalization within tumors, which includes reducing vascular density, increasing pericyte coverage, and enhancing perfusion. This, in turn, suppresses the level of hypoxia in tumor tissue, improves the metabolic profile of the tumor microenvironment, and enhances the delivery and efficacy of exogenous therapeutic agents (41). The anti-tumor mechanisms of the combination of anlotinib and anti-PD-L1 drugs are multifaceted. Firstly, anlotinib induces the expression of PD-L1 through a JAK2 and STAT3 signaling pathway mediated by autocrine IL-6 secretion; the combined use of anlotinib and anti-PD-L1 drugs increases the infiltration of interferon-γ positive CD8+ T cells and natural killer (NK) cells and reduces the number of regulatory T cells (Tregs) and myeloid-derived suppressor cells (MDSCs), thereby producing a significant synergistic therapeutic effect (21). Secondly, anlotinib can promote the normalization of tumor vasculature through CD4+ T cells, transforming the immunosuppressive tumor microenvironment into an immunostimulatory one, inhibiting tumor growth, and preventing systemic immunosuppression. Moreover, the combination of anlotinib and a PD-1 checkpoint inhibitor can counteract the immunosuppression caused by the upregulation of PD-L1 after monotherapy, prolong the period of vessel normalization, and ultimately induce tumor regression (42). Thirdly, anlotinib enhances the infiltration of CD8+ T cells by inducing CCL5, improving the efficacy of PD-1/PD-L1 inhibitors (43). Fourthly, anlotinib may enhance the efficacy of anti-PD-1 antibody therapy by promoting the apoptosis of tumor-associated fibroblasts through the inhibition of the AKT pathway (44). A case of esophageal neuroendocrine carcinoma showed a 29-month response with anlotinib and camrelizumab (19). Another case of esophageal neuroendocrine carcinoma showed a 16-month response to anlotinib with tislelizumab (20). The case reported here harbored an immune “cold tumor” with negative PD-L1 expression. When octreotide plus everolimus proved ineffective, the treatment regimen of octreotide plus IBI-318 plus anlotinib produced a certain efficacy, which showed that the synergistic effect of IBI-318 and anlotinib might have a certain impact in patients with cold NETs.

The case reported here suggests that SSAs and antiangiogenic TKI drugs combined with PD-1/PD-L1 inhibitors might be a feasible option for NETs with unknown primary lesions that failed first-line treatment, but their efficacy and safety need to be further explored in a large sample population. A recent multicenter retrospective study of 304 patients with metastatic NETs showed a median PFS of 7.9 months with capecitabine combined with temozolomide as a second-line treatment (45). In contrast, the second-line combination therapy used here achieved a PFS of at least 11 months (treatment is still ongoing).

However, there are limitations to this approach. Currently, there are no anti-PD-1/PD-L1 bispecific antibodies on the market, which may limit the broader application of this drug combination. It may be necessary to replace IBI-318 with other immunotherapeutic agents in future studies. Furthermore, the absence of standardized second-line treatment protocols for G2 NETs with unknown primary origins necessitates further prospective studies to establish effective and safe treatment regimens.

## 4 Conclusion

Octreotide plus IBI318 plus anlotinib achieved benefits in a patient with advanced NETs of unknown primary lesions after first-line treatment failure. This case suggests that combining SSAs, TKI, and PD-1/PD-L1 inhibitors might be an alternative regimen for patients with advanced, well-differentiated NETs whose primary tumor failed first-line treatment. Nevertheless, prospective trials are needed to verify the efficacy and safety of this treatment regimen.

## Data Availability

The original contributions presented in the study are included in the article/supplementary material. Further inquiries can be directed to the corresponding author/s.
